# Multiple refractory intracellular pathogen infections in a human immunodeficiency virus-negative patient with anti-interferon-γ autoantibodies: a case report

**DOI:** 10.1186/s12879-023-08404-8

**Published:** 2023-07-26

**Authors:** Hongxia Wang, Rong Lei, Yang Ji, Wei Xu, Keke Zhang, Xiang Guo

**Affiliations:** 1 Respiratory and Critical Care Medicine, The University of Hong Kong - Shenzhen Hospital, 1, Haiyuan 1St Road, Futian District, Shenzhen, Guangdong China; 2 Shenzhen, China

**Keywords:** *Talaromyces marneffei*, *Mycobacterium kansasii*, Anti-interferon-γ autoantibodies, Listeriosis

## Abstract

**Background:**

The clinical presentation of adult-onset immunodeficiency with anti-interferon (IFN)-γ autoantibodies with intracellular pathogens can be highly variable, which can lead to misdiagnosis during the early stage of disease.

**Case presentation:**

We report a complex case of a 54-year-old Chinese male who was human immunodeficiency virus-negative. He had a presence of anti-IFN-γ autoantibodies and suffered from various intracellular pathogenic infections. The patient was admitted to our hospital for the first time in July 2016 with severe pneumonia, and he experienced multiple pneumonia infections between 2017 and 2019. In March 2019, the patient was hospitalized due to pulmonary lesions and multiple-bone destruction. During hospitalization, the patient was confirmed to have disseminated *Talaromyces marneffei* infection and was successfully treated with antifungal therapy for 1 year. In June 2021, *Mycobacterium kansasii* infection was detected by positive culture and progressive bone destruction. A high concentration of anti-IFN-γ antibodies was observed in the patient’s serum. In addition, *Listeria monocytogenes* was isolated by blood culture, and the presence of *L. monocytogenes* in cerebrospinal fluid was confirmed by next-generation sequencing. Following anti-non-tuberculous mycobacteria (NTM) therapy and anti-bacterial therapy, the patient’s symptoms, pulmonary lesions, and bone destruction gradually improved.

**Conclusions:**

Although the clinical presentation of adult-onset immunodeficiency with anti-IFN-γ autoantibodies can be highly variable, the diagnosis should be considered if patients suffer from unexplained repeated bacterial or opportunistic infections.

Conventional and advanced molecular testing should be used, as needed, for microbiological diagnoses among this special immunodeficient population.

## Introduction

Interferon (IFN) mainly emerges from T helper 1 cells, CD8^+^ T cells, and natural killer cells, and IFN-gamma (IFN-γ)/interleukin (IL)-12 pathways play a crucial role in the host defense against intracellular pathogens.

- 1. Anti-IFN-gamma autoantibodies. We appreciate the inclusion of a reference for the method used to detect these Auto-Abs. However other details are necessary:
  Authors are invited to specifiy in the text the unit of measure of these Auto-Abs. According to the manufactured protocol used for the detection of anti-IFN-gamma autoantibodies what threshold is reported beyond which a sample can be considered positive?
  Authors are invited to specify in the text whether these anti-IFN-gamma autoantibodies are binding antibodies or neutralizing antibodies in order to understand their real biological significance in the context of NTM infections. To help you: binding antobodies bind to different antigenic epitopes of the IFN molecule, some of them being not involved in activating interferon receptors; so far, no clear biological/clinical functions have been attributed to them. Neutralizing antibodies are a subset of binding antibodies which react with the biological active sites of the IFN molecules, thus preventing the interaction of IFN with its receptor.
- 2. In the hospitalization of 2021 Authors suspected T. marneffei infection (and specific treatment was administered to the subject) but from blood culture, patient was positive to L. monocytogenes. Therefore, the patient was positive also to T. marneffei or not? Authors are invited to clarify this detail in the text. Moreover, in the Discussion, Authors indicate: “The present report is the first to describe a patient with anti-IFN-γ autoantibodies who was not only infected with T. marneffei and M. kansasii…” (line 195–196). It is correct to indicate that anti-IFN-gamma Autoabs are detected in the moment of T. marneffei infection? of anti-IFN-γ autoantibodies affect the natural inflammatory response to infection. *Talaromyces marneffei* (*T. marneffei*) and non-tuberculous mycobacteria are strongly associated with a high anti-IFN-γ autoantibody titer in human immunodeficiency virus (HIV)-negative patients [1–3]. It is rare to encounter patients who are co-infected with disseminated *T. marneffei* and disseminated mycobacterium pathogens; however, mortality is often high in affected patients [4–14]. As a result of its non-specific symptoms and rare occurrence, it may be easily misdiagnosed in the early stage. Here, we report a complex case of a 54-year-old HIV-negative Chinese male with a presence anti-IFN-γ autoantibody titer who was not only infected with disseminated *T. marneffei* and mycobacterium pathogens, but also with *Listeria monocytogenes* (*L. monocytogenes*) in the blood and cerebrospinal fluid (CSF). We herein describe our observations in a HIV-negative patient with anti-IFN-γ autoantibodies to improve the diagnosis and treatment of simultaneous disseminated infections with intracellular pathogens. This study was approved by the Ethics Committee at the University of Hong Kong—Shenzhen Hospital and complied with the principles of the Declaration of Helsinki.

## Case report

**Fig. 1:**
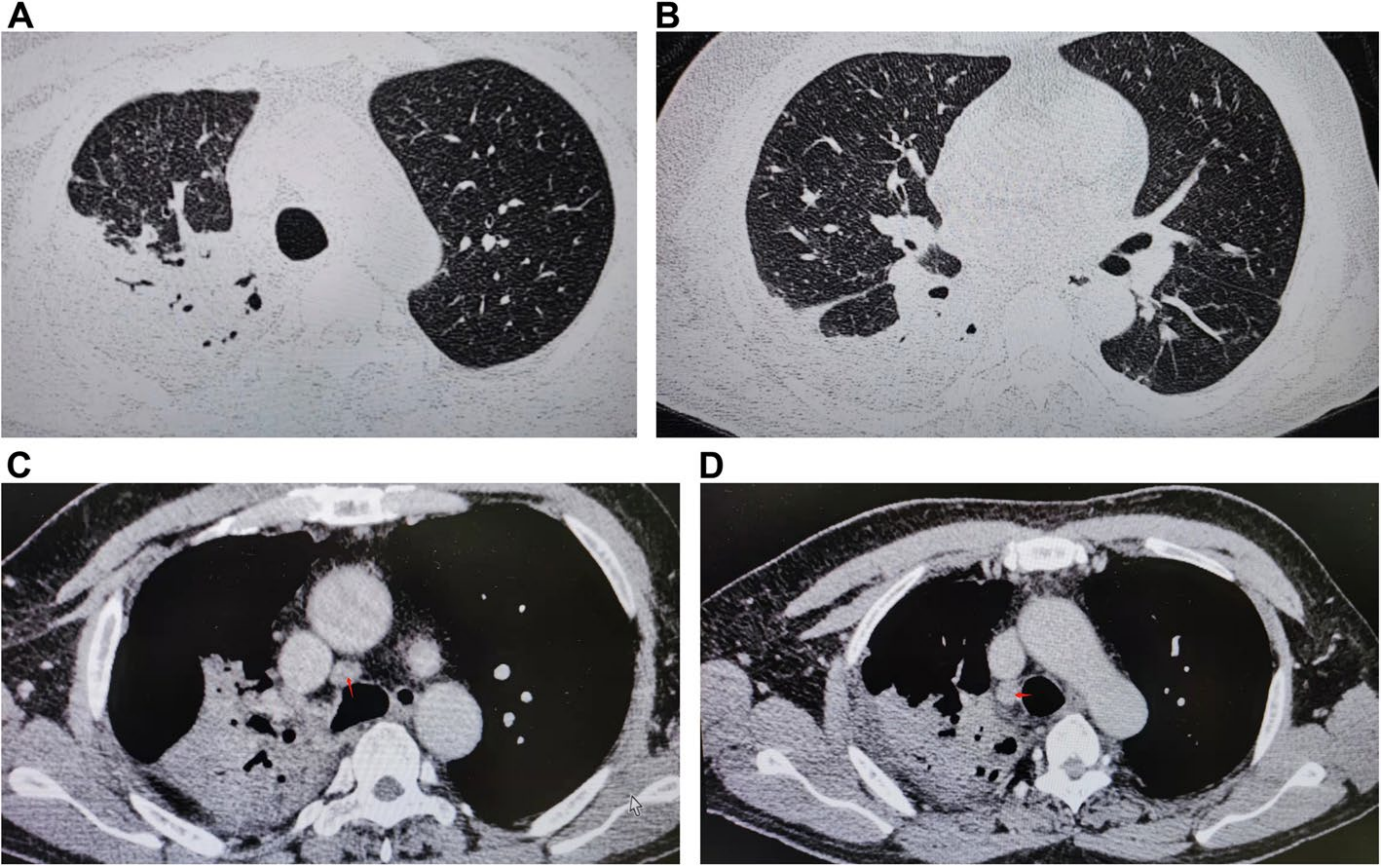
**A**–**D** Chest CT image showing patchy infiltration in the right upper lobe and lower lobe in the lung window, as well as lymph node enlargement in the mediastinal window (July 2016). CT, computed tomography

The patient was a 54-year-old male who was first hospitalized at the respiratory department of our clinic on July 17, 2016, due to pulmonary lesions. The patient’s general condition was poor, and he had experienced cough, hemoptysis, and dyspnea for 2 weeks. Right lung breathing sounds disappeared during chest auscultation. The patient’s medical history and family history were not remarkable. He reported no history of smoking, exposure to occupational hazards, or other unhealthy habits. Blood tests showed a white blood cell count of 24.5 × 109/L (normal range, 3.89–9.93 × 109/L) with a high C-reactive protein (CRP) concentration of 267.9 mg/L. HIV serology was negative. In addition, *Aspergillus* antigen, β-D-glucan, tumor markers, and IFN-γ release assays were within normal ranges. Sputum cultures showed *Burkholderia cepacia* and *Staphylococcus aureus*. Therefore, we performed chest computed tomography (CT) and bronchoscopy. Chest CT showed consolidation of the right upper lung and lymph node enlargement (Fig. 1A–D). There were no pathological macroscopic findings during bronchoscopy. Bronchoalveolar lavage fluid (BALF) was negative for bacteria. The patient was treated with meropenem for 2 weeks, followed by piperacillin/tazobactam for 2 weeks. The patient’s condition improved, and he was discharged. The patient was again admitted to hospital on two separate occasions for lung lobe pneumonia in May 2017 and May 2018.

**Fig. 2:**
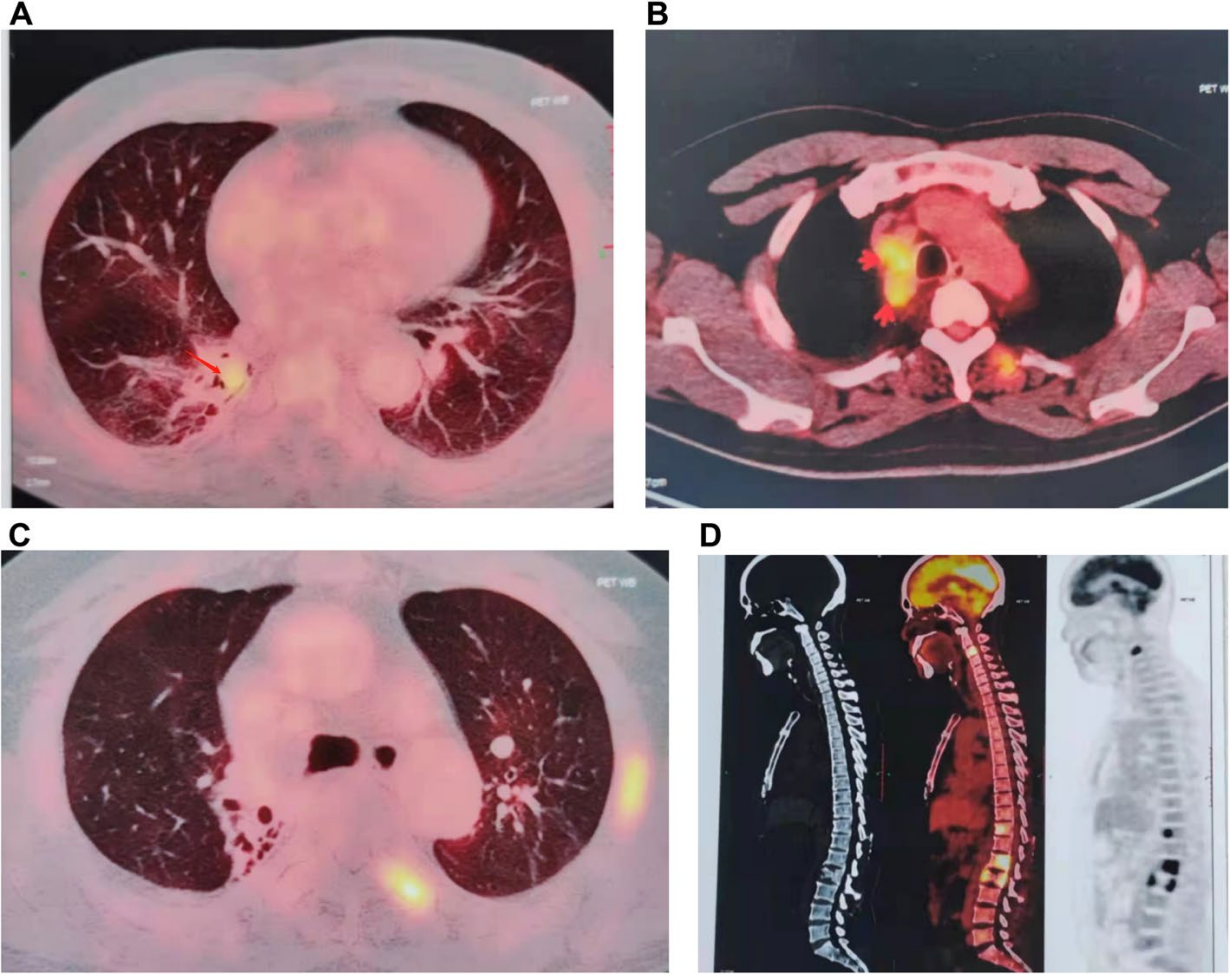
(**A**) PET-CT image (October 2018) displaying alveolar consolidation in the right upper lobe. **B** Multiple hypermetabolically enlarged lymph nodes can be seen in the right hilar and mediastinum. **C**–**D** The right clavicle, ribs, and multiple pyramids in the neck, chest, and waist showed a strong radioactive concentration shadow. CT, computed tomography; PET, positron emission tomography

In October 2018, the patient demonstrated bone fracture and multiple-bone destruction on lumbar CT at another hospital. Positron emission tomography (PET)-CT displayed right hilar hypermetabolic lesions. Multiple hypermetabolically enlarged lymph nodes were observed in the right hilar and mediastinum (Fig. 2A–B). Multiple ribs on both sides and multiple pyramids in the neck, chest, and waist showed high-level radioactive concentration shadows (Fig. 2C–D). As a result, the patient underwent bronchoscopy and L2 vertebra resection biopsy. Unfortunately, the result of pulmonary biopsy was still negative. However, the pathological results of the lumbar spine demonstrated skeletal lesions, which were suspected as multiple myeloma. Four subsequent bone marrow biopsies were performed, which excluded multiple myeloma. From October 2018 to March 2019, the patient’s condition was stable, and he only presented with mild cough and back pain after lumbar instrumentation.

**Fig. 3:**
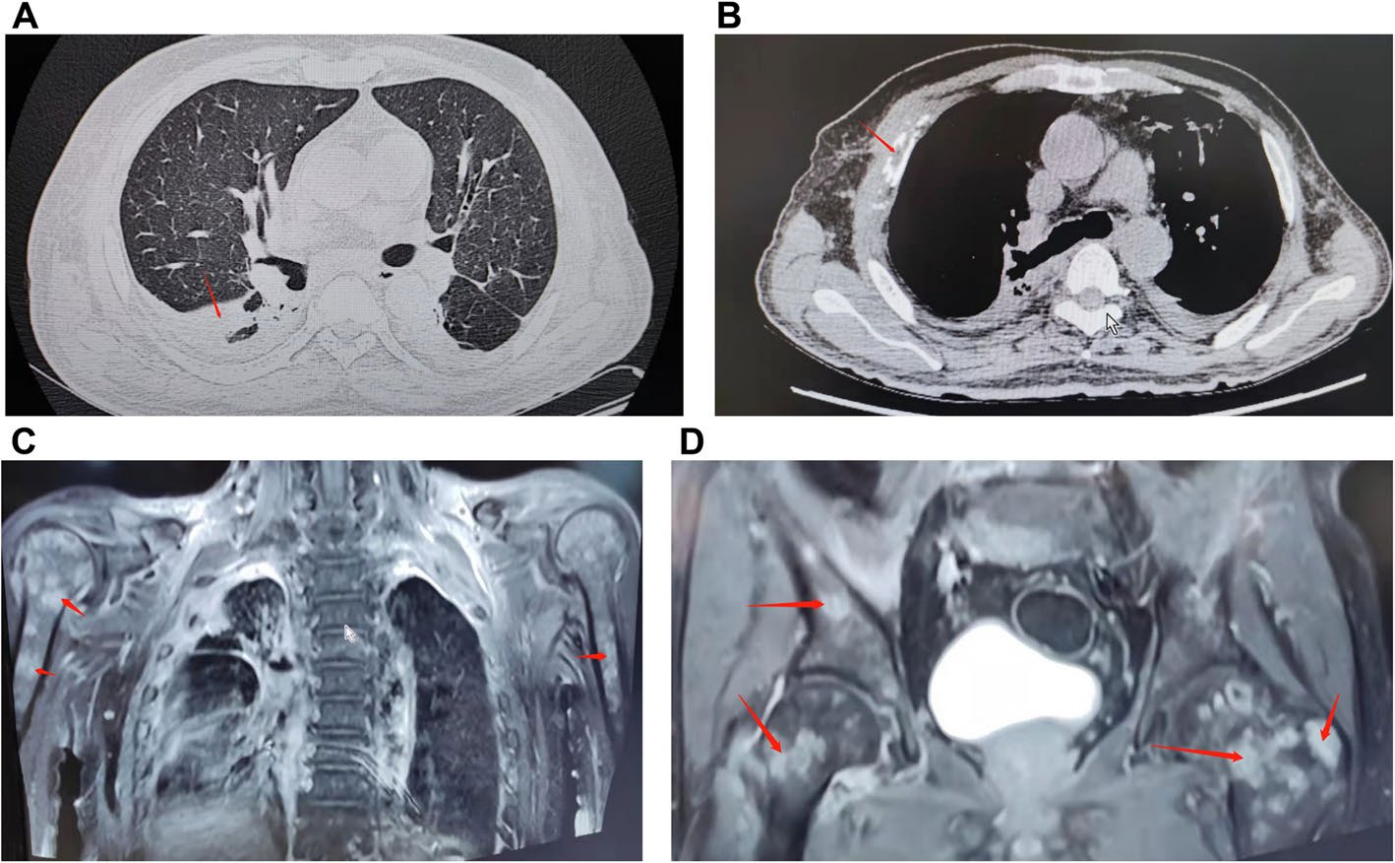
**A**–**B** Chest CT image showing alveolar consolidation in the left upper lobe (arrows) and chest wall rib destruction (March 2019) (arrows). **C**–**E** Spinal and pelvic MRI images showing bone destruction in the spine, sternum, scapula, humerus, and pelvis (arrows). CT, computed tomography; MRI, magnetic resonance imaging;

**Fig. 4:**
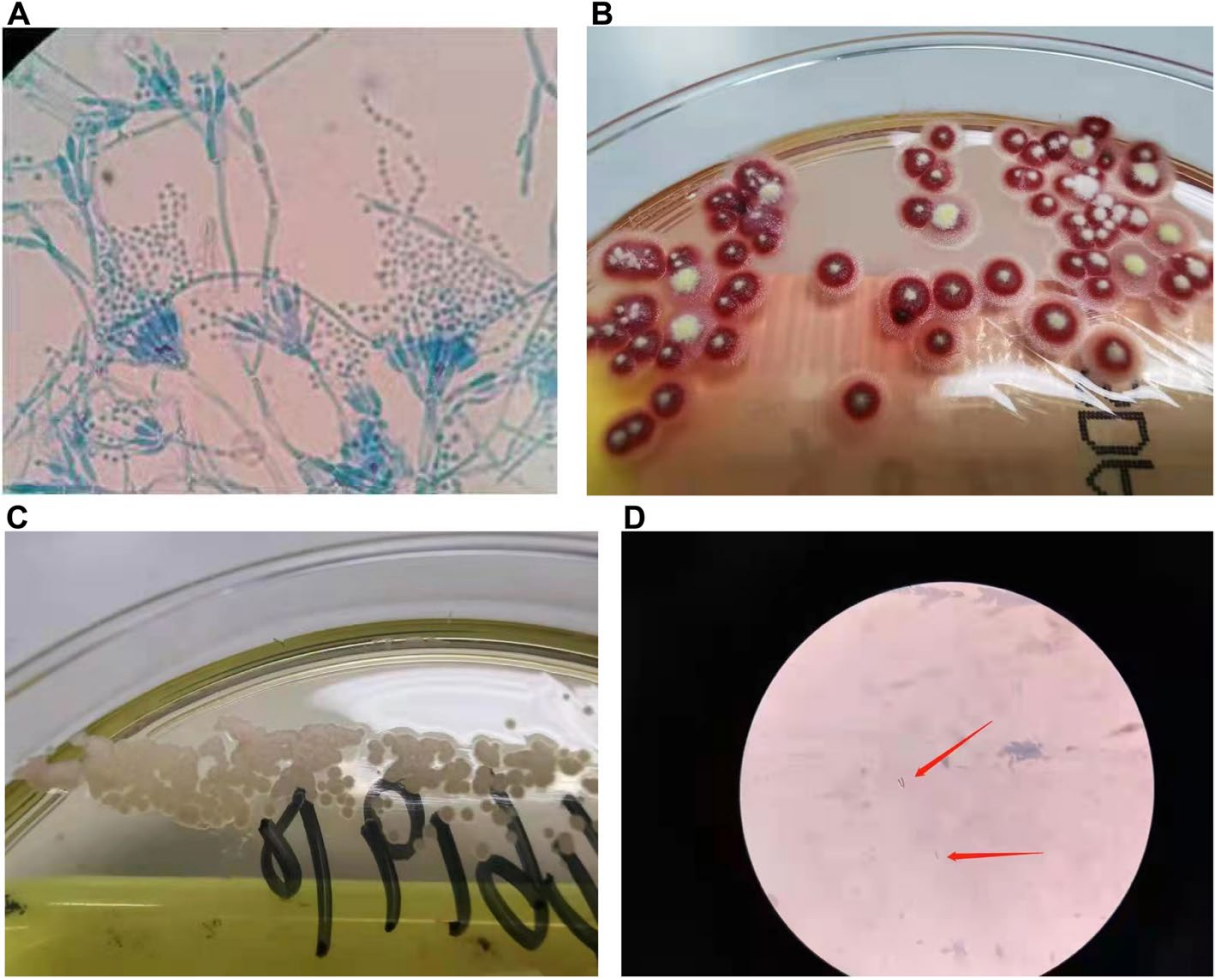
**A**–**B** Cervical lymph node sample on Sabouraud agar at 25 °C showing a granular colony of *Talaromyces marneffei* (*T. marneffei*) with a characteristic soluble red pigment that diffused into the incubation agar. **C** Cervical lymph node sample cultured on Sabouraud agar at 35 °C showing rounded, grayish-white *T. marneffei* colonies. **D** Acid-fast bacilli can be seen in the bone marrow smear and sacral puncture tissue

**Fig. 5:**
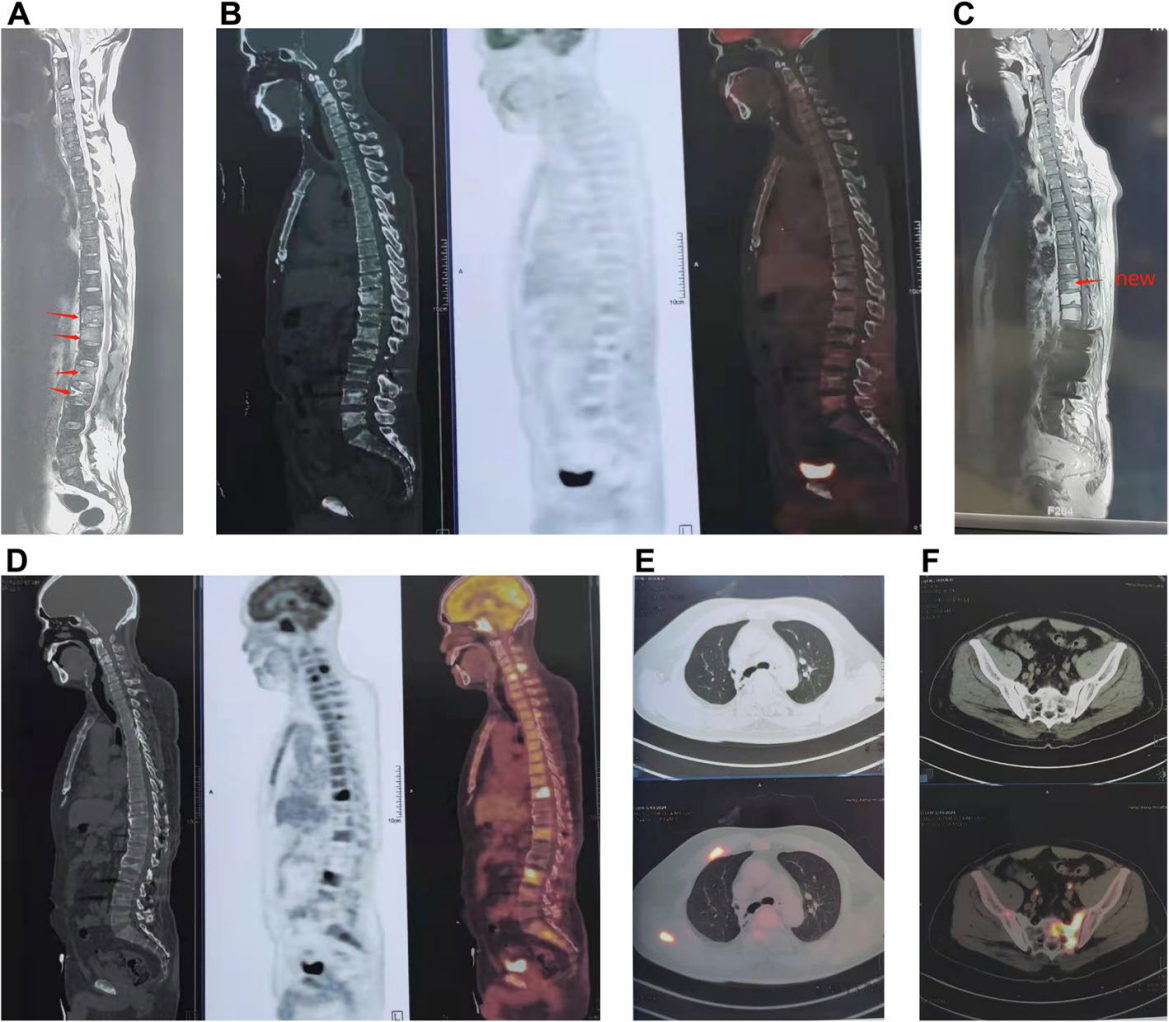
**A** PET image showing that the hypermetabolic lesions were significantly reduced in numbers compared with those on PET performed in October 2018 after 3 months of anti-fungal treatment (June 2019). **B** Spinal MR image showing new abnormal lesions at the level of the T9 vertebra. **D**–**E** Compared with PET performed in June 2019, PET performed in June 2021 showed that hypermetabolic lesions increased both in size and numbers significantly in the spine, rib, and pelvis. CT, computed tomography; MR, magnetic resonance; PET, positron emission tomography

On March 7, 2019, the patient was hospitalized again because his condition worsened. He experienced high fever, dyspnea, and chest pain. The physical examination showed cachexia and several cervical lymph nodes in the right supraclavicular fossa with a maximum diameter of 2 cm and a clear boundary. Blood tests showed an elevated white blood cell count (19.01 × 10^9^/L) with a high CRP concentration of 162.8 mg/L. Blood and sputum cultures, including acid-fast bacteria, were negative. Chest CT still revealed a right lung lesion with pleural effusion and chest wall rib destruction (Fig. 3A–B). Whole-spine and pelvic magnetic resonance imaging (MRI) revealed multiple bone changes in the spine, sternum, scapula, humerus, and pelvis (Fig. 3C–D). We performed cervical lymph node biopsy, which revealed *T. marneffei* (Fig. 4A–C). Pathological findings revealed neutrophil necrosis and plasma cell and lymphocyte infiltration. Abscess formation and nodular changes with central necrosis were also observed. Acid-fast stain, periodic acid–Schiff, and Gömöri methenamine silver staining were negative for pathogens. The patient was treated with intravenous meropenem and liposomal amphotericin B (5 mg/kg/day) for 2 weeks. To analyze whether *T. marneffei* was the cause of the pulmonary lesions and multiple skeletal lesions, we performed bronchoscopy and thoracic vertebra biopsy again. However, the bacterial, fungal, and mycobacterial cultures were all negative. The patient continued to take voriconazole (200 mg every 12 h) for 12 months after discharge. The patient’s condition improved without fever or respiratory symptoms, but he still suffered from bone pain. PET showed that the hypermetabolic lesions were significantly reduced compared with those on PET in October 2018 after 3 months of anti-fungal treatment (Fig. 5A).

**Fig. 6:**
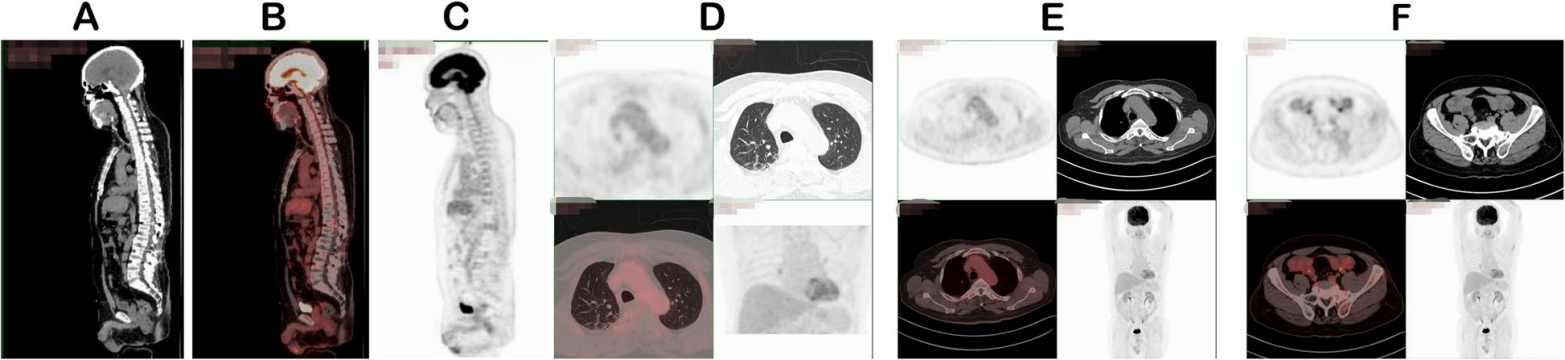
**A**–**F** PET image showing that the hypermetabolic lesions were significantly reduced compared with those on PET performed in June 2021 after 1 year of anti-NTM treatment (May 2022). NTM, non-tuberculous mycobacteria; PET, positron emission tomography

In June 2021, the patient was hospitalized again because of fever with persistent headache and lower back pain. The patient’s white blood cell count was 24.47 × 10^9^/L, and the CRP concentration was 282.42 mg/L. Serum cryptococcal capsular antigen tests were negative. (1, 3)-β-D glucan, galactomannan, blood CD4^+^ T cell counts, immunoglobulin (Ig) (including IgM, IgA, and IgG), complement C3, and complement C4 were within normal reference ranges. We tested the patient’s immune function and found a presence concentration of anti-IFN-γ autoantibodies. The anti-IFN-gamma autoantibodies are neutralizing antibodies which are a subset of binding antibodies which react with the biological active sites of the IFN molecules, thus preventing the interaction of IFN with its receptor The detection method of anti-IFN-γ autoantibodies is the same as the description in Tang’s study [11]. This method only detects anti-IFN-γautoantibodies but not their neutralizing activity, which is a limitation of this study.Spinal MRI showed new abnormal lesions at the level of the T9 vertebra (Fig. 5B). We suspected *T. marneffei* infection relapse, so we treated the patient with intravenous voriconazole. On the second day after admission, *L. monocytogenes* was isolated from blood culture. *L. monocytogenes i*s the causative bacterial species of listeriosis, which is a food-borne infection that is usually caused by consumption of unpasteurized milk and dairy products. Our patient denied a history of an unclean diet and presented with fever and headache without influenza-like symptoms or diarrhea. We suspected that the patient may have unknowingly consumed food that was past it expiry date. We performed lumbar puncture and sent the CSF for examination and next-generation sequencing, which confirmed that the patient had meningitis caused by *L. monocytogenes*. The CSF was colorless with a cell count of 387 × 10^6^/L (82% monocytes, 10% lymphocytes, 8% neutrophils), a protein concentration of 373 mg/L, and a glucose concentration of 2.8 mmol/L. Gram smear showed suspicious Gram-negative bacilli, but the culture was negative. Meanwhile, piperacillin/tazobactam was changed to intravenous ampicillin for anti-bacterial therapy. PET-CT showed that the patient had multiple nodular abnormally high radiation uptake lesions in the bilateral iliac bones, bilateral acetabulum, and left ischium. Compared with PET performed in June 2019, PET performed in June 2021 showed a significant increase in both In the size and number of the hypermetabolic lesions in the spine, ribs, and pelvis (Fig. 5C–F). The sacral lesion was biopsied by fine-needle aspiration. Bone marrow puncture fluid was cultured as *Mycobacterium kansasii* (*M. kansasii*) (Fig. 4D). Pathological findings demonstrated a large number of neutrophils and lymphocyte and plasmocyte infiltration. Necrosis and epithelioid tissue hyperplasia were also observed. There was no evidence that patient was infected by T. marneffei. Anti-bacterial therapy was changed to intravenous meropenem (2 g every 8 h), tigecycline (50 mg every 12 h), amikacin (500 mg every 24 h), linezolid (600 mg every 24 h), and oral ethambutol (1 g every 24 h). After discharge, the patient took oral clarithromycin (500 mg every 12 h), rifampicin (300 mg every 12 h), ethambutol (1 g every 24 h), and linezolid (600 mg every 24 h) for 1 year. During the 1-year follow-up period after discharge, the PET re-examination results showed a significant improvement in bone metabolism (Fig. 6A–F). Considering the bone destruction, we advised the patient to continue taking his medication for 6 months. As of July 8 2022, the patient had no discomfort and his inflammatory markers were normal, but the IFN-γ autoantibody concentration was high, warranting further follow-up.

## Discussion

Adult-onset immunodeficiency with anti-IFN-γ autoantibodies was first described in 2004 in patients with mycobacterial infection [1]. However, the specific mechanism of how anti-IFN-γ autoantibodies lead to immunodeficiency is still unclear. Previous studies have suggested that patients have high serum anti-IFN-γ autoantibody titers, which can inhibit IFN-γ-induced CD4^+^ T cell STAT-1 phosphorylation and IL-12 production, leading to serious dysfunction in the T helper 1 cell response [15, 16], which is the cause of multiple severe, and sometimes fatal, intracellular pathogen infections in HIV-negative patients. In addition, immunodeficiency may be caused by genetic factor susceptibility. Recent studies have shown that the human leukocyte antigen class II alleles DRB and DQB are related to anti-IFN-γ autoantibodies associated with immunodeficiency [17, 18].

*T. marneffei* is an opportunistic fungus that is mainly observed in HIV-positive patients in Southeast Asia. However, in recent years, the number of patients without HIV who are diagnosed with *T. marneffei* infection is increasing. In a recent study, all patients with HIV demonstrated positive *T. marneffei* cultures, while 63.6% of immunocompromised patients without HIV and 28% of immunocompetent patients with negative culture results were diagnosed by metagenomic next-generation sequencing [19]. It is easily misdiagnosed in patients without HIV infection, and our report fully demonstrates this point. Our patient had obvious bone destruction in October 2018, but he was not diagnosed with *T. marneffei* infection until March 2019. During this period, the patient underwent surgical biopsy of lumbar bone and repeated tracheoscopy, but all of the culture results were negative. Interestingly, after 1 year of antifungal treatment and 1 year of drug withdrawal, MRI showed that rib and sternum bone destruction had improved, but a new abnormal lesion was detected at the level of the T9 vertebra. We suspected *T. marneffei* infection relapse, so we treated the patient with intravenous voriconazole. Fortunately, we insisted on performing another bone puncture on the new lesions due to the high anti-IFN-γ autoantibody titer, and the patient was finally confirmed to have *M. kansasii* infection in bone marrow puncture fluid. Disseminated NTM infections are the most significant phenotype associated with anti-IFN-γ autoantibodies. It has been reported that approximately 81% of patients with disseminated NTM with no obvious immunodeficiency and normal CD4 lymphocyte counts are positive for anti-IFN-γ autoantibodies [2]. Osteolytic destruction in the present case was caused by both NTM and *T. marneffei*, which illustrates the complexity of our case and suggests that we should have considered the possibility of infection with other pathogens as the disease progressed after treatment.

The present report is the first to describe a patient with anti-IFN-γ autoantibodies who was not only infected with *T. marneffei and M. kansasii,* but who also had listeriosis caused by *L. monocytogenes*, which is a Gram-positive bacillus and facultative intracellular bacterium [20]. The most common forms of the infection are maternal–neonatal infection, bacteremia, and neurolisteriosis, particularly in immunocompromised hosts [21]. Listeriosis is a food-borne infection usually caused by consumption of unpasteurized milk and dairy products. Our patient did not appear to have an unclean diet and presented with fever and headache without influenza-like symptoms or diarrhea. Although blood culture was positive and CSF culture was negative, the presence of *L. monocytogenes* in the CSF was confirmed by next-generation sequencing. Our case demonstrates that next-generation sequencing can improve the diagnosis of some culture-negative pathogen infections. The present report is different from previous reports in that our patient suffered from several potentially fatal bacterial infections in the lungs before *T. marneffei* and NTM infection were detected. Clinicians are often consciously aware of immunosuppression in patients with *T. marneffei*, NTM, or opportunistic infections, but they rarely pay attention to the immunity of patients with bacterial infections only. However, the present case suggests that repeated bacterial infections may also result from immunosuppression. Anti-IFN-γ autoantibody testing is not routine in the clinic; however, our case suggests that anti-IFN-γ autoantibody testing should be recommended if patients suffer from repeated bacterial infections. Thus far, there is no standardized method to treat patients with adult-onset immunodeficiency with anti-IFN-γ autoantibodies, except for anti-infective treatment. Only three small-sample studies have reported that rituximab can eliminate anti-IFN-γ autoantibodies by targeting B cells, which can achieve sustained remission of infection [22–24]. Our patient remained in a good overall condition after 12 months of anti-mycobacterial treatment. To date, he has not undergone rituximab treatment, but he has taken some Chinese herbal medicines, such as *Tripterygium wilfordii*, which is usually used to cure rheumatoid arthritis, nephrotic syndrome, and systemic lupus erythematosus by doctors in traditional Chinese medicine. *Tripterygium wilfordii* has a variety of pharmacological and immunomodulatory effects, including anti-cancer, anti-inflammatory, immunosuppressive, anti-fertility, anti-viral, and anti-microbial effects [25, 26]; however, its specific effects still need further evaluation.

## Conclusions

Although the clinical presentation of adult-onset immunodeficiency with anti-IFN-γ autoantibodies can be highly variable, the diagnosis should be considered if patients suffer from unexplained repeated bacterial or opportunistic infections. Conventional and advanced molecular testing should be used, as needed, for microbiological diagnoses among this special immunodeficient population.

## Data Availability

The datasets used or analysed during the current study available from the corresponding author on reasonable request.
